# 1-(3,5-Diethyl-1*H*-pyrazol-1-yl)-3-phenyl­isoquinoline

**DOI:** 10.1107/S1600536810028059

**Published:** 2010-07-21

**Authors:** F. Nawaz Khan, P. Manivel, S. Mohana Roopan, Venkatesha R. Hathwar, Mehmet Akkurt

**Affiliations:** a Organic and Medicinal Chemistry Research Laboratory, Organic Chemistry Division, School of Advanced Sciences, VIT University, Vellore 632 014, Tamil Nadu, India; b Solid State and Structural Chemistry Unit, Indian Institute of Science, Bangalore 560 012, Karnataka, India; c Department of Physics, Faculty of Arts and Sciences, Erciyes University, 38039 Kayseri, Turkey

## Abstract

In the title mol­ecule, C_22_H_21_N_3_, the isoquinoline ring is almost planar [maximum deviation = 0.046 (1) Å] and makes dihedral angles of 52.01 (4) and 14.61 (4)° with the pyrazole and phenyl rings, respectively. The phenyl ring and the pyrazole ring are twisted by 44.20 (6)° with respect to each other. The terminal C atoms of both of the ethyl groups attached to the pyrazole ring are disordered over two sites with occupancy ratios of 0.164 (7):0.836 (7) and 0.447 (16):0.553 (16). A weak intra­molecular C—H⋯N contact may influence the mol­ecular conformation. The crystal structure is stabilized by C—H⋯π contacts involving the phenyl and pyrazole rings, and by π–π stacking inter­actions involving the pyridine and benzene rings [centroid–centroid distance = 3.5972 (10) Å].

## Related literature

For the biological actvity of pyrazoles, see: Huang *et al.* (1996 ▶); Li *et al.* (2005 ▶); Patel *et al.* (1990 ▶); Zhao *et al.* (2001 ▶). For the crystal structures of pyrazoles, see: Manivel *et al.* (2009 ▶); Khan *et al.* (2010*a* ▶,*b* ▶,*c* ▶). For the crystal structure of an isoquinazole, see: Hathwar *et al.* (2008 ▶).
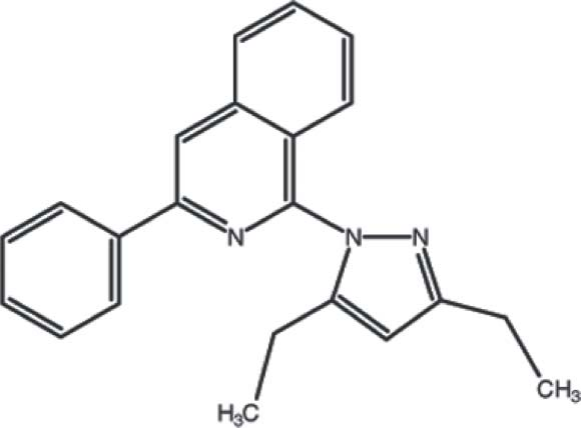


## Experimental

### 

#### Crystal data

C_22_H_21_N_3_
*M* *_r_* = 327.42
Monoclinic, 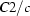

*a* = 16.0736 (17) Å
*b* = 20.819 (2) Å
*c* = 10.8579 (11) Å
β = 91.071 (3)°
*V* = 3632.8 (6) Å^3^
*Z* = 8
Mo *K*α radiation
μ = 0.07 mm^−1^
*T* = 295 K
0.25 × 0.21 × 0.15 mm

#### Data collection

Oxford Xcalibur Eos (Nova) CCD detector diffractometer
17963 measured reflections
3389 independent reflections
2379 reflections with *I* > 2σ(*I*)
*R*_int_ = 0.029

#### Refinement

*R*[*F* ^2^ > 2σ(*F* ^2^)] = 0.044
*wR*(*F* ^2^) = 0.130
*S* = 1.06
3389 reflections
264 parameters
10 restraints
H-atom parameters constrained
Δρ_max_ = 0.15 e Å^−3^
Δρ_min_ = −0.15 e Å^−3^

### 

Data collection: *CrysAlis PRO CCD* (Oxford Diffraction, 2009 ▶); cell refinement: *CrysAlis PRO CCD*; data reduction: *CrysAlis PRO RED* (Oxford Diffraction, 2009 ▶); program(s) used to solve structure: *SHELXS97* (Sheldrick, 2008 ▶); program(s) used to refine structure: *SHELXL97* (Sheldrick, 2008 ▶); molecular graphics: *ORTEP-3 for Windows* (Farrugia, 1997 ▶); software used to prepare material for publication: *WinGX* (Farrugia, 1999 ▶), *PARST* (Nardelli, 1983 ▶) and *PLATON* (Spek, 2009 ▶).

## Supplementary Material

Crystal structure: contains datablocks global, I. DOI: 10.1107/S1600536810028059/su2194sup1.cif

Structure factors: contains datablocks I. DOI: 10.1107/S1600536810028059/su2194Isup2.hkl

Additional supplementary materials:  crystallographic information; 3D view; checkCIF report

## Figures and Tables

**Table 1:** Hydrogen-bond geometry (Å, °) *Cg*1 is the centroid of the N2/N3/C16–C18 ring.

*D*—H⋯*A*	*D*—H	H⋯*A*	*D*⋯*A*	*D*—H⋯*A*
C3—H3⋯N3	0.93	2.50	3.001 (2)	114
C14—H14⋯*Cg*1^i^	0.93	2.88	3.755 (2)	158

Symmetry code: (i) 
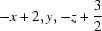
.

**Fig. 1.**
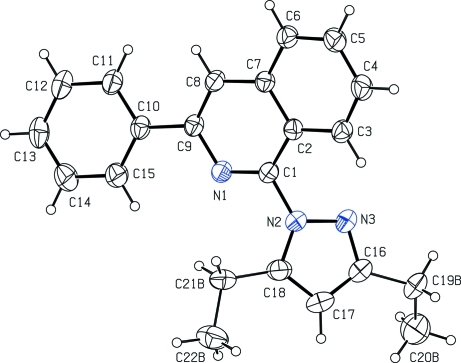
A view of the molecular structure of the title molecule with the atom numbering scheme and displacement ellipsoids drawn at the 30% probability level. Only the major components of the disordered ethyl goups are shown.

**Fig. 2.**
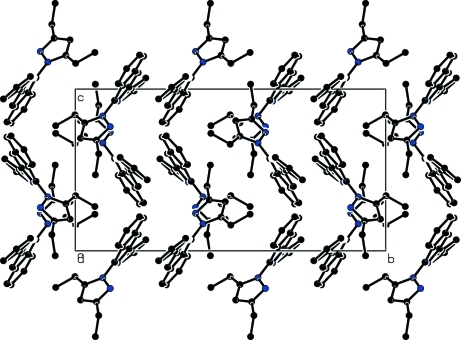
The crystal packing diagram of the title compound viewed along the c axis. Only the major components of the disordered ethyl goups are shown (H-atoms have been omitted for clarity).

## supplementary crystallographic information

### Comment

Pyrazole and its derivatives are a class of important five-membered heterocycle compounds with two adjacent nitrogen atoms. During the past years considerable evidence has been accumulated to demonstrate the biological efficacy of pyrazole derivatives, including antibacterial (Patel *et al.*, 1990), antifungal (Zhao *et al.*, 2001), herbicidal (Li *et al.*, 2005), insectcidal (Huang *et al.*, 1996) and other biological activities. A number of pyrazole-containing compounds have been successfully commercialized, such as the blockbuster drugs Viagra, Celebrex, and Acomplia. In view of the diverse applications of this class of compounds, and continuing our research on the synthesis and crystal structure analysis of similar compounds (Manivel *et al.*, 2009; Khan *et al.*, 2010*a*,*b*,*c*; Hathwar *et al.*, 2008), we report herein on the crystal structure of the new title isoquinoline pyrazole.

In the title molecule (Fig. 1) the isoquinoline ring (N1/C1–C9) is essentially planar, with a maximum deviation of 0.046 (1) Å for atom C1 and makes dihedral angles of 52.01 (4) and 14.61 (4) ° with the pyrazole (N2/N3/C16–C18) and phenyl (C10–C15) rings, respectively. The phenyl ring and the pyrazole ring are twisted by 44.20 (6)° with respect to each other. There are weak intramolecular C—H···N contacts which may influence the molecular conformation of the molecule (Table 1).

The crystal packing of the title compound is illustrated in Fig. 2. The crystal structure is stabilized by C—H···π contacts involving both the phenyl and pyrazole rings (Table 1), and by π-π stacking interactions between the pyridine and benzene rings; *Cg*2···*Cg*3^i^ (symmetry code: (i) = 3/2 - *x*, 1/2 - *y*, 1 - *z*), with a centroid-to-centroid distance of 3.5972 (10) Å [*Cg*2 and *Cg*3 are the centroids of the pyridine (N1/C1/C2/C7–C9) and benzene (C2–C7) rings, respectively, of the isoquinoline group].

### Experimental

1-(3-phenylisoquinolin-1-yl)hydrazine (2.35 g, 10 mmol) and heptane-3,5-dione (1.28 g, 10 mmol) were dissolved in ethanol (30 ml). The solution was heated for 12 h under a nitrogen atmosphere. The reaction was quenched with water; the product was extracted with ethyl acetate. This phase was then washed with water, dried, concentrated and purified by column chromatography to yield a white powder. Crystals, suitable for X-ray diffraction analysis, were obtained upon recrystallization from dichloromethane.

### Refinement

The C-atoms of the two ethyl groups (C19/C20 and C21/C22) are positionally disordered. The ratio of the site occupancies of the major and minor components (A:B) are 0.164 (7):0.836 (7) and 0.447 (16):0.553 (16), respectively. The positional and thermal displacement parameters for atom C20A were refined isotropically with *U*_iso_(C)= 0.168 (10) Å^-2^. H-atoms were placed in calculated positions and were included in the refinement in the riding model approximation: C-H = 0.93, 0.97 and 0.96 Å for CH, CH_2_ and CH_3_ H-atoms, respectively, with *U*_iso_(H) = k × *U*_eq_(C), where k = 1.5 for CH_3_ H-atoms and 1.2 for all other H-atoms.

### Crystal data

**Table:**

C_22_H_21_N_3_	*F*(000) = 1392
*M**_r_* = 327.42	*D*_x_ = 1.197 Mg m^−^^3^
Monoclinic, *C*2/*c*	Mo *K*α radiation, λ = 0.71073 Å
Hall symbol: -C 2yc	Cell parameters from 1236 reflections
*a* = 16.0736 (17) Å	θ = 1.9–20.4°
*b* = 20.819 (2) Å	µ = 0.07 mm^−^^1^
*c* = 10.8579 (11) Å	*T* = 295 K
β = 91.071 (3)°	Block, colourless
*V* = 3632.8 (6) Å^3^	0.25 × 0.21 × 0.15 mm
*Z* = 8	

### Data collection

**Table:**

Oxford Xcalibur Eos (Nova) CCD detector diffractometer	2379 reflections with *I* > 2σ(*I*)
Radiation source: Enhance (Mo) X-ray Source	*R*_int_ = 0.029
graphite	θ_max_ = 25.5°, θ_min_ = 1.6°
ω scans	*h* = −19→19
17963 measured reflections	*k* = −25→24
3389 independent reflections	*l* = −11→13

### Refinement

**Table:**

Refinement on *F*^2^	Secondary atom site location: difference Fourier map
Least-squares matrix: full	Hydrogen site location: inferred from neighbouring sites
*R*[*F*^2^ > 2σ(*F*^2^)] = 0.044	H-atom parameters constrained
*wR*(*F*^2^) = 0.130	*w* = 1/[σ^2^(*F*_o_^2^) + (0.0667*P*)^2^ + 0.6171*P*] where *P* = (*F*_o_^2^ + 2*F*_c_^2^)/3
*S* = 1.06	(Δ/σ)_max_ < 0.001
3389 reflections	Δρ_max_ = 0.15 e Å^−^^3^
264 parameters	Δρ_min_ = −0.15 e Å^−^^3^
10 restraints	Extinction correction: *SHELXL97* (Sheldrick, 2008), FC^*^=KFC[1+0.001XFC^2^Λ^3^/SIN(2Θ)]^-1/4^
Primary atom site location: structure-invariant direct methods	Extinction coefficient: 0.0037 (5)

### Special details

**Table:**

Geometry. Bond distances, angles *etc*. have been calculated using the rounded fractional coordinates. All su's are estimated from the variances of the (full) variance-covariance matrix. The cell e.s.d.'s are taken into account in the estimation of distances, angles and torsion angles
Refinement. Refinement on *F*^2^ for ALL reflections except those flagged by the user for potential systematic errors. Weighted *R*-factors *wR* and all goodnesses of fit *S* are based on *F*^2^, conventional *R*-factors *R* are based on *F*, with *F* set to zero for negative *F*^2^. The observed criterion of *F*^2^ > σ(*F*^2^) is used only for calculating -*R*-factor-obs *etc*. and is not relevant to the choice of reflections for refinement. *R*-factors based on *F*^2^ are statistically about twice as large as those based on *F*, and *R*-factors based on ALL data will be even larger.

### Fractional atomic coordinates and isotropic or equivalent isotropic displacement parameters (Å^2^)

**Table:**

	*x*	*y*	*z*	*U*_iso_*/*U*_eq_	Occ. (<1)
N1	0.85495 (7)	0.13529 (6)	0.57519 (11)	0.0503 (4)	
N2	0.74093 (8)	0.08848 (6)	0.66680 (12)	0.0543 (4)	
N3	0.67528 (9)	0.11249 (7)	0.73249 (14)	0.0693 (5)	
C1	0.77419 (9)	0.12658 (7)	0.57018 (13)	0.0469 (5)	
C2	0.71957 (8)	0.15187 (7)	0.47772 (13)	0.0452 (5)	
C3	0.63356 (9)	0.13837 (7)	0.46657 (15)	0.0560 (5)	
C4	0.58747 (10)	0.16524 (8)	0.37411 (17)	0.0641 (6)	
C5	0.62327 (10)	0.20691 (9)	0.28960 (16)	0.0651 (6)	
C6	0.70590 (9)	0.22017 (8)	0.29663 (15)	0.0581 (5)	
C7	0.75675 (9)	0.19240 (7)	0.39001 (13)	0.0467 (5)	
C8	0.84337 (9)	0.20270 (7)	0.39824 (14)	0.0513 (5)	
C9	0.89063 (9)	0.17344 (7)	0.48754 (13)	0.0474 (5)	
C10	0.98262 (9)	0.18012 (8)	0.49702 (13)	0.0517 (5)	
C11	1.02486 (10)	0.22715 (9)	0.43174 (15)	0.0619 (6)	
C12	1.11065 (10)	0.23165 (10)	0.43877 (16)	0.0692 (7)	
C13	1.15577 (11)	0.18965 (10)	0.51142 (17)	0.0719 (7)	
C14	1.11534 (11)	0.14448 (11)	0.57762 (19)	0.0815 (8)	
C15	1.02943 (11)	0.13958 (10)	0.57140 (17)	0.0711 (7)	
C16	0.65965 (12)	0.06812 (10)	0.81487 (18)	0.0749 (7)	
C17	0.71487 (13)	0.01688 (9)	0.80421 (18)	0.0781 (7)	
C18	0.76646 (11)	0.03038 (8)	0.70880 (16)	0.0642 (6)	
C19B	0.5866 (3)	0.0755 (2)	0.8999 (4)	0.0936 (16)	0.836 (7)
C20B	0.6107 (2)	0.0648 (2)	1.0302 (3)	0.1248 (16)	0.836 (7)
C21B	0.8444 (7)	−0.0011 (5)	0.6591 (13)	0.074 (3)	0.553 (16)
C22B	0.8635 (6)	−0.0631 (3)	0.7263 (6)	0.092 (2)	0.553 (16)
C20A	0.5561 (14)	0.0333 (11)	0.945 (3)	0.168 (10)*	0.164 (7)
C21A	0.8228 (9)	−0.0155 (7)	0.6459 (16)	0.084 (4)	0.447 (16)
C22A	0.8969 (7)	−0.0292 (9)	0.7276 (10)	0.130 (5)	0.447 (16)
C19A	0.6194 (13)	0.0870 (14)	0.936 (2)	0.139 (15)	0.164 (7)
H8	0.86860	0.22980	0.34200	0.0620*	
H11	0.99490	0.25600	0.38270	0.0740*	
H6	0.72930	0.24780	0.23950	0.0700*	
H3	0.60850	0.11110	0.52260	0.0670*	
H4	0.53110	0.15570	0.36700	0.0770*	
H5	0.59040	0.22570	0.22810	0.0780*	
H17	0.71640	−0.01980	0.85310	0.0940*	
H19C	0.56360	0.11830	0.89080	0.1120*	0.836 (7)
H19D	0.54360	0.04490	0.87620	0.1120*	0.836 (7)
H20D	0.63820	0.02400	1.03830	0.1870*	0.836 (7)
H20E	0.56190	0.06510	1.07980	0.1870*	0.836 (7)
H20F	0.64780	0.09830	1.05710	0.1870*	0.836 (7)
H21C	0.83650	−0.00980	0.57190	0.0890*	0.553 (16)
H21D	0.89110	0.02810	0.66870	0.0890*	0.553 (16)
H22D	0.87290	−0.05430	0.81220	0.1380*	0.553 (16)
H22E	0.91240	−0.08230	0.69270	0.1380*	0.553 (16)
H22F	0.81730	−0.09200	0.71670	0.1380*	0.553 (16)
H12	1.13790	0.26320	0.39420	0.0830*	
H13	1.21350	0.19220	0.51510	0.0860*	
H14	1.14570	0.11650	0.62780	0.0980*	
H15	1.00290	0.10850	0.61800	0.0850*	
H19A	0.65910	0.08660	1.00400	0.1650*	0.164 (7)
H19B	0.59300	0.12880	0.93020	0.1650*	0.164 (7)
H20A	0.52560	0.02950	0.86890	0.2520*	0.164 (7)
H20B	0.51840	0.04290	1.01040	0.2520*	0.164 (7)
H20C	0.58430	−0.00630	0.96300	0.2520*	0.164 (7)
H21A	0.84090	0.00290	0.56880	0.1010*	0.447 (16)
H21B	0.79320	−0.05510	0.62770	0.1010*	0.447 (16)
H22A	0.92540	0.01020	0.74650	0.1950*	0.447 (16)
H22B	0.93390	−0.05790	0.68620	0.1950*	0.447 (16)
H22C	0.87880	−0.04880	0.80260	0.1950*	0.447 (16)

### Atomic displacement parameters (Å^2^)

**Table:**

	*U*^11^	*U*^22^	*U*^33^	*U*^12^	*U*^13^	*U*^23^
N1	0.0470 (7)	0.0557 (7)	0.0482 (7)	−0.0026 (6)	0.0025 (6)	0.0012 (6)
N2	0.0520 (8)	0.0535 (7)	0.0577 (8)	−0.0024 (6)	0.0082 (6)	0.0090 (6)
N3	0.0609 (9)	0.0738 (9)	0.0739 (10)	0.0036 (7)	0.0235 (8)	0.0165 (8)
C1	0.0466 (9)	0.0467 (8)	0.0477 (8)	−0.0033 (6)	0.0064 (7)	−0.0005 (6)
C2	0.0424 (8)	0.0442 (8)	0.0491 (8)	−0.0010 (6)	0.0030 (7)	−0.0040 (6)
C3	0.0480 (9)	0.0535 (9)	0.0665 (10)	−0.0083 (7)	0.0045 (8)	0.0003 (8)
C4	0.0432 (9)	0.0714 (11)	0.0773 (12)	−0.0063 (8)	−0.0060 (8)	−0.0004 (9)
C5	0.0521 (10)	0.0808 (12)	0.0620 (10)	0.0031 (8)	−0.0076 (8)	0.0060 (9)
C6	0.0487 (9)	0.0719 (10)	0.0536 (9)	−0.0018 (8)	0.0000 (8)	0.0078 (8)
C7	0.0451 (8)	0.0500 (8)	0.0449 (8)	−0.0005 (6)	0.0021 (7)	−0.0019 (6)
C8	0.0448 (8)	0.0628 (9)	0.0466 (8)	−0.0065 (7)	0.0053 (7)	0.0054 (7)
C9	0.0434 (8)	0.0565 (9)	0.0424 (8)	−0.0033 (7)	0.0034 (7)	−0.0021 (7)
C10	0.0447 (9)	0.0683 (10)	0.0421 (8)	−0.0025 (7)	0.0027 (7)	−0.0056 (7)
C11	0.0463 (9)	0.0835 (12)	0.0561 (10)	−0.0064 (8)	0.0049 (7)	0.0028 (8)
C12	0.0506 (10)	0.0963 (14)	0.0611 (10)	−0.0153 (9)	0.0103 (8)	−0.0099 (10)
C13	0.0421 (9)	0.1042 (15)	0.0694 (12)	−0.0041 (10)	0.0004 (9)	−0.0195 (11)
C14	0.0522 (11)	0.1047 (15)	0.0868 (14)	0.0059 (10)	−0.0162 (10)	0.0089 (12)
C15	0.0517 (10)	0.0917 (13)	0.0698 (11)	−0.0019 (9)	−0.0046 (9)	0.0154 (10)
C16	0.0676 (12)	0.0825 (13)	0.0752 (12)	−0.0030 (10)	0.0185 (10)	0.0225 (10)
C17	0.0911 (14)	0.0724 (12)	0.0713 (12)	−0.0081 (10)	0.0117 (11)	0.0263 (10)
C18	0.0743 (12)	0.0581 (10)	0.0602 (10)	0.0020 (8)	0.0034 (9)	0.0118 (8)
C19B	0.073 (3)	0.117 (3)	0.092 (2)	0.000 (2)	0.034 (2)	0.0342 (19)
C20B	0.135 (3)	0.159 (3)	0.082 (2)	0.045 (3)	0.042 (2)	0.017 (2)
C21B	0.085 (5)	0.056 (4)	0.082 (4)	0.005 (3)	0.008 (4)	0.022 (3)
C22B	0.122 (5)	0.067 (3)	0.086 (3)	0.021 (3)	0.000 (3)	0.010 (3)
C21A	0.093 (7)	0.061 (6)	0.099 (6)	0.011 (5)	0.021 (6)	0.010 (5)
C22A	0.107 (6)	0.132 (10)	0.151 (7)	0.046 (6)	−0.026 (5)	−0.020 (7)
C19A	0.058 (12)	0.21 (3)	0.15 (3)	−0.034 (13)	0.004 (12)	0.093 (19)

### Geometric parameters (Å, °)

**Table:**

N1—C1	1.3108 (18)	C3—H3	0.9300
N1—C9	1.3733 (19)	C4—H4	0.9300
N2—N3	1.379 (2)	C5—H5	0.9300
N2—C1	1.4271 (19)	C6—H6	0.9300
N2—C18	1.354 (2)	C8—H8	0.9300
N3—C16	1.313 (3)	C11—H11	0.9300
C1—C2	1.422 (2)	C12—H12	0.9300
C2—C3	1.4137 (19)	C13—H13	0.9300
C2—C7	1.413 (2)	C14—H14	0.9300
C3—C4	1.357 (2)	C15—H15	0.9300
C4—C5	1.395 (2)	C17—H17	0.9300
C5—C6	1.357 (2)	C19A—H19A	0.9700
C6—C7	1.414 (2)	C19A—H19B	0.9700
C7—C8	1.410 (2)	C19B—H19C	0.9700
C8—C9	1.364 (2)	C19B—H19D	0.9700
C9—C10	1.487 (2)	C20A—H20A	0.9600
C10—C11	1.393 (2)	C20A—H20B	0.9600
C10—C15	1.381 (2)	C20A—H20C	0.9600
C11—C12	1.383 (2)	C20B—H20D	0.9600
C12—C13	1.375 (3)	C20B—H20F	0.9600
C13—C14	1.357 (3)	C20B—H20E	0.9600
C14—C15	1.385 (3)	C21A—H21B	0.9700
C16—C17	1.394 (3)	C21A—H21A	0.9700
C16—C19B	1.515 (5)	C21B—H21C	0.9700
C16—C19A	1.53 (2)	C21B—H21D	0.9700
C17—C18	1.368 (3)	C22A—H22A	0.9600
C18—C21B	1.522 (12)	C22A—H22B	0.9600
C18—C21A	1.491 (15)	C22A—H22C	0.9600
C19A—C20A	1.52 (3)	C22B—H22D	0.9600
C19B—C20B	1.477 (5)	C22B—H22E	0.9600
C21A—C22A	1.499 (19)	C22B—H22F	0.9600
C21B—C22B	1.511 (13)		
			
N1···C21B	2.988 (11)	H3···N3	2.5000
N1···C21A	3.275 (15)	H3···N2	2.6600
N3···C3	3.001 (2)	H3···H20B^ix^	2.5100
N1···H21D	2.5200	H3···H20C^iii^	2.3100
N1···H15	2.4800	H4···H20E^ix^	2.4800
N1···H21A	2.7700	H6···H8	2.5100
N2···H3	2.6600	H6···C13^x^	3.0200
N2···H14^i^	2.9100	H8···C11	2.6800
N3···H3	2.5000	H8···H6	2.5100
C1···C6^ii^	3.515 (2)	H8···H11	2.1400
C2···C7^ii^	3.564 (2)	H8···C12^vi^	3.0700
C2···C8^ii^	3.472 (2)	H11···C8	2.6800
C3···N3	3.001 (2)	H11···H8	2.1400
C4···C11^ii^	3.587 (2)	H12···H19B^vii^	2.4000
C5···C9^ii^	3.483 (2)	H14···N2^i^	2.9100
C6···C9^ii^	3.597 (2)	H14···C18^i^	2.8700
C6···C1^ii^	3.515 (2)	H15···H21D	2.5200
C7···C8^ii^	3.575 (2)	H15···N1	2.4800
C7···C7^ii^	3.395 (2)	H17···C20A	2.9900
C7···C2^ii^	3.564 (2)	H17···C22B	2.9000
C8···C7^ii^	3.575 (2)	H17···H20D	2.5600
C8···C22B^iii^	3.473 (7)	H17···C2^v^	3.0600
C8···C2^ii^	3.472 (2)	H17···C3^v^	3.0700
C9···C6^ii^	3.597 (2)	H17···H20C	2.4700
C9···C5^ii^	3.483 (2)	H19B···H12^xi^	2.4000
C11···C4^ii^	3.587 (2)	H19B···C12^xi^	2.9200
C20A···C20A^iv^	2.59 (4)	H20A···C20A^iv^	2.7600
C21A···N1	3.275 (15)	H20A···H20B^iv^	2.1300
C21B···N1	2.988 (11)	H20B···H3^ix^	2.5100
C22A···C22A^i^	3.341 (16)	H20B···C20A^iv^	2.0500
C22B···C8^v^	3.473 (7)	H20B···H20C^iv^	1.8500
C1···H21C	3.0100	H20B···H20A^iv^	2.1300
C1···H21A	2.7900	H20B···H20B^iv^	1.8900
C1···H21D	2.9700	H20C···C20A^iv^	2.5500
C2···H17^iii^	3.0600	H20C···H3^v^	2.3100
C3···H17^iii^	3.0700	H20C···H20B^iv^	1.8500
C3···H20C^iii^	2.8600	H20C···C3^v^	2.8600
C7···H22F^iii^	2.9900	H20C···H17	2.4700
C8···H22F^iii^	3.0600	H20C···C17	2.7800
C8···H11	2.6800	H20D···C17	2.8500
C11···H8	2.6800	H20D···H17	2.5600
C12···H8^vi^	3.0700	H20D···C18^v^	2.9700
C12···H19B^vii^	2.9200	H20E···H4^ix^	2.4800
C13···H6^viii^	3.0200	H21A···C1	2.7900
C17···H22C	2.9700	H21A···N1	2.7700
C17···H20C	2.7800	H21C···C1	3.0100
C17···H20D	2.8500	H21D···C1	2.9700
C17···H22F	2.9700	H21D···H15	2.5200
C17···H22D	2.9400	H21D···N1	2.5200
C18···H20D^iii^	2.9700	H22A···C22A^i^	2.9800
C18···H14^i^	2.8700	H22A···H22A^i^	2.4000
C20A···H17	2.9900	H22B···H22B^i^	2.5100
C20A···H20C^iv^	2.5500	H22B···C22A^i^	2.9200
C20A···H20B^iv^	2.0500	H22C···C17	2.9700
C20A···H20A^iv^	2.7600	H22D···C17	2.9400
C22A···H22B^i^	2.9200	H22F···C17	2.9700
C22A···H22A^i^	2.9800	H22F···C7^v^	2.9900
C22B···H17	2.9000	H22F···C8^v^	3.0600
			
C1—N1—C9	118.51 (12)	C12—C11—H11	120.00
N3—N2—C1	118.67 (12)	C11—C12—H12	120.00
N3—N2—C18	112.33 (13)	C13—C12—H12	120.00
C1—N2—C18	128.97 (13)	C12—C13—H13	120.00
N2—N3—C16	104.80 (14)	C14—C13—H13	120.00
N1—C1—N2	115.51 (13)	C13—C14—H14	120.00
N1—C1—C2	125.16 (13)	C15—C14—H14	120.00
N2—C1—C2	119.33 (13)	C10—C15—H15	120.00
C1—C2—C3	125.21 (13)	C14—C15—H15	119.00
C1—C2—C7	115.69 (12)	C16—C17—H17	126.00
C3—C2—C7	119.09 (13)	C18—C17—H17	126.00
C2—C3—C4	120.05 (14)	C16—C19A—H19A	112.00
C3—C4—C5	121.12 (15)	C16—C19A—H19B	112.00
C4—C5—C6	120.32 (16)	C20A—C19A—H19A	112.00
C5—C6—C7	120.60 (15)	C20A—C19A—H19B	112.00
C2—C7—C6	118.77 (13)	H19A—C19A—H19B	110.00
C2—C7—C8	118.53 (13)	C16—C19B—H19D	109.00
C6—C7—C8	122.70 (13)	C20B—C19B—H19C	109.00
C7—C8—C9	120.88 (14)	C16—C19B—H19C	109.00
N1—C9—C8	121.11 (13)	H19C—C19B—H19D	108.00
N1—C9—C10	115.71 (12)	C20B—C19B—H19D	109.00
C8—C9—C10	123.18 (13)	C19A—C20A—H20B	109.00
C9—C10—C11	121.61 (14)	C19A—C20A—H20A	110.00
C9—C10—C15	120.90 (14)	H20A—C20A—H20B	110.00
C11—C10—C15	117.50 (14)	H20A—C20A—H20C	110.00
C10—C11—C12	120.97 (16)	C19A—C20A—H20C	109.00
C11—C12—C13	120.21 (17)	H20B—C20A—H20C	109.00
C12—C13—C14	119.47 (17)	C19B—C20B—H20D	109.00
C13—C14—C15	120.81 (19)	C19B—C20B—H20E	110.00
C10—C15—C14	121.01 (18)	H20E—C20B—H20F	110.00
N3—C16—C17	110.58 (17)	C19B—C20B—H20F	109.00
N3—C16—C19B	120.3 (2)	H20D—C20B—H20E	109.00
N3—C16—C19A	119.7 (11)	H20D—C20B—H20F	109.00
C17—C16—C19B	129.1 (2)	C18—C21A—H21B	110.00
C17—C16—C19A	123.3 (10)	C22A—C21A—H21A	110.00
C16—C17—C18	107.55 (17)	C22A—C21A—H21B	110.00
N2—C18—C17	104.74 (15)	C18—C21A—H21A	110.00
N2—C18—C21B	120.8 (5)	H21A—C21A—H21B	108.00
N2—C18—C21A	127.0 (6)	C18—C21B—H21C	109.00
C17—C18—C21B	134.0 (5)	C22B—C21B—H21C	109.00
C17—C18—C21A	126.7 (6)	C22B—C21B—H21D	109.00
C16—C19A—C20A	99.3 (19)	C18—C21B—H21D	109.00
C16—C19B—C20B	112.1 (3)	H21C—C21B—H21D	108.00
C18—C21A—C22A	109.5 (12)	C21A—C22A—H22B	109.00
C18—C21B—C22B	111.0 (9)	C21A—C22A—H22C	109.00
C2—C3—H3	120.00	C21A—C22A—H22A	110.00
C4—C3—H3	120.00	H22A—C22A—H22C	109.00
C3—C4—H4	119.00	H22B—C22A—H22C	109.00
C5—C4—H4	119.00	H22A—C22A—H22B	110.00
C4—C5—H5	120.00	C21B—C22B—H22D	109.00
C6—C5—H5	120.00	C21B—C22B—H22E	109.00
C5—C6—H6	120.00	C21B—C22B—H22F	109.00
C7—C6—H6	120.00	H22D—C22B—H22E	109.00
C7—C8—H8	120.00	H22D—C22B—H22F	109.00
C9—C8—H8	120.00	H22E—C22B—H22F	110.00
C10—C11—H11	120.00		
			
C9—N1—C1—N2	−177.66 (12)	C4—C5—C6—C7	−0.5 (3)
C9—N1—C1—C2	2.3 (2)	C5—C6—C7—C2	−1.6 (2)
C1—N1—C9—C8	1.1 (2)	C5—C6—C7—C8	177.46 (16)
C1—N1—C9—C10	−178.72 (13)	C2—C7—C8—C9	1.1 (2)
C1—N2—N3—C16	−178.86 (14)	C6—C7—C8—C9	−177.98 (15)
C18—N2—N3—C16	−0.80 (19)	C7—C8—C9—N1	−2.8 (2)
N3—N2—C1—N1	128.12 (15)	C7—C8—C9—C10	177.06 (14)
N3—N2—C1—C2	−51.86 (19)	N1—C9—C10—C11	−166.37 (14)
C18—N2—C1—N1	−49.6 (2)	N1—C9—C10—C15	13.7 (2)
C18—N2—C1—C2	130.45 (17)	C8—C9—C10—C11	13.8 (2)
N3—N2—C18—C17	0.31 (19)	C8—C9—C10—C15	−166.20 (16)
N3—N2—C18—C21B	−172.9 (6)	C9—C10—C11—C12	−178.17 (16)
C1—N2—C18—C17	178.12 (15)	C15—C10—C11—C12	1.8 (3)
C1—N2—C18—C21B	5.0 (6)	C9—C10—C15—C14	178.09 (17)
N2—N3—C16—C17	1.0 (2)	C11—C10—C15—C14	−1.9 (3)
N2—N3—C16—C19B	−175.9 (2)	C10—C11—C12—C13	−0.4 (3)
N1—C1—C2—C3	174.67 (15)	C11—C12—C13—C14	−1.1 (3)
N1—C1—C2—C7	−3.9 (2)	C12—C13—C14—C15	1.0 (3)
N2—C1—C2—C3	−5.4 (2)	C13—C14—C15—C10	0.5 (3)
N2—C1—C2—C7	176.11 (13)	N3—C16—C17—C18	−0.8 (2)
C1—C2—C3—C4	−179.85 (15)	C19B—C16—C17—C18	175.7 (3)
C7—C2—C3—C4	−1.4 (2)	N3—C16—C19B—C20B	−130.4 (3)
C1—C2—C7—C6	−178.91 (14)	C17—C16—C19B—C20B	53.4 (4)
C1—C2—C7—C8	2.0 (2)	C16—C17—C18—N2	0.3 (2)
C3—C2—C7—C6	2.5 (2)	C16—C17—C18—C21B	172.1 (7)
C3—C2—C7—C8	−176.61 (14)	N2—C18—C21B—C22B	175.9 (5)
C2—C3—C4—C5	−0.7 (2)	C17—C18—C21B—C22B	5.1 (12)
C3—C4—C5—C6	1.6 (3)		

Symmetry codes: (i) −*x*+2, *y*, −*z*+3/2; (ii) −*x*+3/2, −*y*+1/2, −*z*+1; (iii) *x*, −*y*, *z*−1/2; (iv) −*x*+1, −*y*, −*z*+2; (v) *x*, −*y*, *z*+1/2; (vi) −*x*+2, *y*, −*z*+1/2; (vii) *x*+1/2, −*y*+1/2, *z*−1/2; (viii) *x*+1/2, −*y*+1/2, *z*+1/2; (ix) −*x*+1, *y*, −*z*+3/2; (x) *x*−1/2, −*y*+1/2, *z*−1/2; (xi) *x*−1/2, −*y*+1/2, *z*+1/2.

### Hydrogen-bond geometry (Å, °)

**Table:**

Cg1 is the centroid of the N2/N3/C16–C18 ring.

**Table:**

*D*—H···*A*	*D*—H	H···*A*	*D*···*A*	*D*—H···*A*
C3—H3···N3	0.93	2.50	3.001 (2)	114
C15—H15···N1	0.93	2.48	2.807 (2)	101
C14—H14···Cg1^i^	0.93	2.88	3.755 (2)	158

Symmetry codes: (i) −*x*+2, *y*, −*z*+3/2.
